# Deep learning-based assessment of coronary artery disease using curved-multiplanar reconstruction: comprehensive evaluation of stenosis, calcification, and plaque

**DOI:** 10.3389/frai.2026.1602119

**Published:** 2026-03-19

**Authors:** Keewon Shin, Namkug Kim, Jonathan A. Leipsic, Ji Ho Lee, Dae-Won Kim

**Affiliations:** 1 Department of Biomedical Engineering, Asan Medical Institute of Convergence Science and Technology, Asan Medical Center, University of Ulsan College of Medicine, Seoul, Republic of Korea; 2 Department of Radiology, Asan Medical Center, College of Medicine, Seoul, Republic of Korea; 3 Department of Radiology, St. Paul’s Hospital, University of British Columbia, Vancouver, BC, Canada; 4 Division of Cardiology, Daejeon St. Mary’s Hospital, College of Medicine, The Catholic University of Korea, Seoul, Republic of Korea

**Keywords:** coronary artery disease, computed tomography angiography, deep learning, stenosis quantification, vulnerable plaque, CAD-RADS 2.0

## Abstract

**Introduction:**

This study developed and validated a deep learning-based framework to detect and quantify coronary artery stenosis, vulnerable plaque, and calcification in Curved-Multiplanar Reconstruction (cMPR) images to support clinical decision-making in coronary artery disease (CAD).

**Methods:**

We analyzed 1,715 patients (5,112 vessels) from 2014 to 2022. Each vessel was reconstructed into a cMPR scan consisting of 13 sequential cross-sectional slices. Using a 2D nnU-Net framework, we developed a Stenosis Segmentation Model (SSM) and a Vulnerable Plaque Segmentation Model (VPSM). A time-independent test set (*n* = 824 patients, 2,437 vessels) was used for unbiased evaluation. Performance was assessed using Dice Similarity Coefficient (DSC), Positive Predictive Value (PPV), and Negative Predictive Value (NPV). For stenosis quantification, Mean Absolute Error (MAE) and Bland–Altman analysis were employed.

**Results:**

The SSM achieved a vessel-level sensitivity of 0.84 and a high NPV of 0.98. The MAE for diameter stenosis was 12.4%, with a mean bias of +1.2% in Bland–Altman analysis, demonstrating robust agreement with expert references across the full test spectrum. For vulnerable plaque detection, the VPSM achieved a sensitivity of 0.80 and an NPV of 0.97 at the slice level. Calcification assessment showed high inter-rater reliability (ICC = 0.84) and substantial agreement with expert visual scoring (Kappa = 0.76).

**Conclusion:**

The proposed automated analysis demonstrated high diagnostic reliability, particularly in its negative predictive power, making it a powerful non-invasive tool for CAD screening. By providing objective quantification of stenosis, calcification, and vulnerable plaques, this method offers a significant advancement in standardized cMPR evaluation in clinical environments.

## 1 Introduction

Coronary artery disease (CAD) stands as a prominent global health challenge, with its effective and timely diagnosis being crucial for preventing critical cardiac outcomes (Knuuti et al., 2020). The emergence of cross-sectional coronary computed tomography angiography (CCTA) as a non-invasive diagnostic tool marks a significant advancement in CAD detection (Abdelrahman et al., 2020). Despite its widespread adoption, the analysis of CCTA images demands considerable expertise. In addition, it is time-intensive and subject to individual interpretation variabilities. These challenges underscore the necessity for more reliable and precise diagnostic methods for CAD assessment using CCTA.

The advent of deep learning (DL) techniques in medical imaging analysis has opened new frontiers in addressing these challenges. DL, a branch of artificial intelligence (AI), has shown remarkable capabilities in interpreting complex patterns from large datasets. Its applications span across various domains, including computer vision, natural language processing, and notably, medical imaging analysis, where it has revolutionized diagnostic procedures (LeCun et al., 2015). In cardiovascular medicine, DL-based models have significantly contributed to the prediction and diagnosis of CAD, enhancing the efficacy of CCTA image interpretation by accurately detecting stenosis, calcification, and vulnerable plaques with high precision and efficiency. This burgeoning research area is pivotal in transforming cardiac diagnostic practices.

Despite its benefits, the clinical utility of CCTA is constrained by factors such as radiation exposure, contrast agent use, and its demanding analysis process. Moreover, clinicians often prefer curved MultiPlanar Reconstruction (cMPR) for its intuitive display of coronary arteries, although it lacks the diagnostic depth of CCTA. Consequently, the development of a specialized screening tool that can leverage cMPR images to analyze major CAD features is imperative. Such a tool can facilitate rapid and straightforward evaluations of CAD severity and risk in CCTA scans.

### 1.1 Related works

In the field of diagnosing CAD with CCTA, significant research efforts have been made. Kirişli et al. (2013) have developed a publicly available evaluation framework for coronary artery stenosis detection and quantification. They found that 11 stenosis algorithms were not sufficiently reliable for clinical practice, with fundamental limitations stemming from CTA imaging constraints including calcification blooming artifacts, motion-related vessel wall blurring, and contrast enhancement heterogeneity. These technical challenges resulted in high false positive rates and inconsistent stenosis quantification across all evaluated methods. Additionally, several recent studies have employed deep learning techniques for calcium scoring in CCTA. van Velzen et al. (2020) have presented a deep learning model for calcium scoring in CCTA with promising results. In the same way, Covas et al. (2022) have reported improvements in DL for imaging coronary atherosclerosis in the heart. Additionally, Yamada et al. (2022) have studied ultra-high-resolution CTA with conventional CTA for quantitative assessment of coronary artery stenosis grading. Lipkin et al. (2022) have compared the accuracy of AI-QCT interpretation with myocardial perfusion imaging for the detection of obstructive stenosis using invasive angiography as a reference standard. Griffin et al. (2023) have used a series of validated convolutional neural networks for AI-guided evaluation of coronary segmentation, lumen wall evaluation, and plaque characterization with CCTA images. The study population consisted of diverse patients with stable chest pain from 23 global sites undergoing CCTA plus quantitative coronary angiography, stress testing, and fractional flow reserve. Their results showed an accuracy of 86%, a sensitivity of 94%, and a specificity of 82% for ≥70% stenosis. Similarly, Choi et al. (2021) have used validated convolutional neural networks for AI-guided evaluation of CCTA images for a population of patients with acute and stable chest pain from three international centers. Their results showed an accuracy of 99.7%, a sensitivity of 90.9%, and a specificity of 99.8% for ≥70% stenosis. Overall, these studies highlight groundbreaking advancements and high potential of AI and DL in CAD diagnosis using CCTA, outperforming traditional diagnostic methods in accuracy and predictive capability.

Recent advances in transformer-based architectures have demonstrated superior performance in coronary analysis. Wang et al. (2023) introduced the DR-LCT-UNet, combining Dense Residual modules with Local Contextual Transformers, achieving 85.8% Dice coefficient with significant improvements over baseline methods. Gerbasi et al. (2024) developed the first Multi-Axis Vision Transformer (MA-ViT) for automated CAD-RADS scoring, demonstrating AUC of 0.87–0.93 while learning from patient-level annotations without requiring fine-grained imaging labels. Commercial AI systems have achieved remarkable clinical validation. The HeartFlow REVEALPLAQUE system demonstrated 95% agreement with IVUS measurements across 258 patients in 15 sites, achieving FDA clearance and Medicare coverage approval. Similarly, Cleerly AI-QCT systems have shown clinical management changes in 57% of patients with multiple FDA breakthrough device designations. Lin et al. (2022) published the most comprehensive international study in The Lancet Digital Health, achieving 100% per-patient sensitivity and 97.5% specificity for ≥70% stenosis detection. However, these high-performance systems typically require extensive computational resources and comprehensive CCTA analysis, limiting their accessibility in resource-constrained clinical environments.

### 1.2 Main contribution

The primary contribution of this study is the implementation of a novel, computationally efficient cMPR-based framework to localize and quantify coronary artery stenosis. While high-end AI-QCT systems offer extensive analysis, they often require significant computational overhead. Our approach optimizes clinical workflow by utilizing cMPR images—a preferred format for clinicians—to provide rapid, objective screening for major CAD features.

Key contributions include:

#### 1.2.1 Robust stenosis quantification

We introduce a method to calculate the Stenosis Regression Index (SRI) and lesion length across the full clinical spectrum, achieving an MAE of 12.4% without biased data filtering.

#### 1.2.2 Clinically validated reliability

Our calcium scoring assistance tool was validated against five expert cardiologists, achieving an intraclass correlation coefficient (ICC) of 0.84, demonstrating “excellent” inter-rater agreement.

#### 1.2.3 High screening utility

By prioritizing negative predictive value (NPV of 0.98), our model ensures reliable exclusion of significant CAD, making it an ideal screening tool in resource-constrained or high-throughput clinical environments.

#### 1.2.4 Plaque characterization

We advanced a model capable of differentiating vulnerable plaques from normal segments, enhancing the diagnostic depth of cMPR-based screening.

## 2 Methods

### 2.1 Dataset

This section delineates the methodology employed in constructing a dataset of cross-sectional CT images from patients diagnosed with stenosis and vulnerable plaque. It also describes the creation of ground truth labels for stenosis severity, vulnerable plaque, and calcification. The studies involving human participants were reviewed and approved by the Institutional Review Board of Daejeon St. Mary’s Hospital, College of Medicine, The Catholic University of Korea (IRB no. DC20RNSI0072). The studies were conducted in accordance with the local legislation and institutional requirements. All patient data were treated with strict confidentiality and analyzed anonymously, and the methodology of this study was aligned with the principles of the Declaration of Helsinki.

#### 2.1.1 Dataset definitions and study population

To ensure consistency in reporting, we categorized our data into four hierarchical units: patients, vessels, cMPR scans, and image slices (Table 1). A total of 1,715 patients at Daejeon St. Mary’s Hospital, South Korea, spanning from April 2014 to February 2022 were included, with each patient providing up to three major coronary vessels (LAD, LCX, RCA). For each vessel, a single curved Multiplanar Reconstruction (cMPR) scan was generated using the CT Cardiac Package from TeraRecon Inc., which was further discretized into 13 sequential cross-sectional slices for deep learning analysis. The dataset was split into development (training and validation) and test sets using a time-independent approach based on the year of examination (2014–2020 for development; 2021-2022 for testing). Within the development set, we implemented a strict patient-level separation with a 4:1 ratio to ensure that slices from the same patient did not appear in both training and validation subsets, thereby preventing data leakage. The exact distribution across each unit and subset is summarized in Table 2.

**Table 1:** Canonical dataset definitions and units of analysis.

Unit term	Clinical/Technical definition	Total count	Role in study
Patient	Individual study participant with an unique ID.	1,715	Unit for patient-level separation to prevent data leakage.
CCTA scan	One complete volumetric CT examination per patient.	1,715	The raw 3D data source for generating cMPR images.
Vessel	Individual coronary artery (LAD, LCX, RCA) analyzed.	5,145	Unit for vessel-level sensitivity/specificity reporting.
cMPR scan	A set of 13 cross-sectional views along a single vessel.	5,145	The primary input unit for the deep learning pipeline.
Slice (image)	A single 2D cross-sectional image (512 × 512 pixels).	66,456	The atomic unit for training/validating segmentation models.

*Due to blurred image, missing slice, file error and prior interventions, 5,112 vessels were analyzed from 1,704 patients.

**Table 2:** Detailed dataset split with patient-level separation.

Dataset subset	Split method	Patients	Vessels	Slices (images)	Split ratio (train:val)
Development set	Random split	891	2,675	34,775	—
Training	Patient-level	713	2,140	27,820	80% (4)
Validation	Patient-level	178	535	6,955	20% (1)
Test set	Time-independent	824	2,437	31,681	
Total		1,715	5,145	66,456	

*Due to blurred image, missing slice, file error and prior interventions, 5,112 vessels were analyzed from 1,704 patients.

Inclusion criteria were: (1) individuals aged 18–75 years, (2) those with a history of coronary artery disease or myocardial infarction, and (3) having no contraindication for cMPR. Exclusion criteria were: (1) images with compromised quality due to motion artifacts, calcification, or inadequate contrast enhancement, and (2) patients with coronary artery bypass grafts or stents. The process involved the following steps:

1) Acquisition of cMPR data from patients diagnosed with stenosis and vulnerable plaque through angiography who underwent cross-sectional CT.
2) Annotation of stenosis severity was restricted to lesions exhibiting more than 40% narrowing, with corresponding ground truth masks applied to images.
3) Identification of vulnerable plaque was confirmed via angiography, followed by marking regions of interest on images.
4) In the absence of ground truth labels for calcification, visual scoring by three experienced cardiac surgeons was employed to assess the calcium score of each image, along with their evaluation of the clinical utility of the final report based on this scoring.

**Figure 1:**
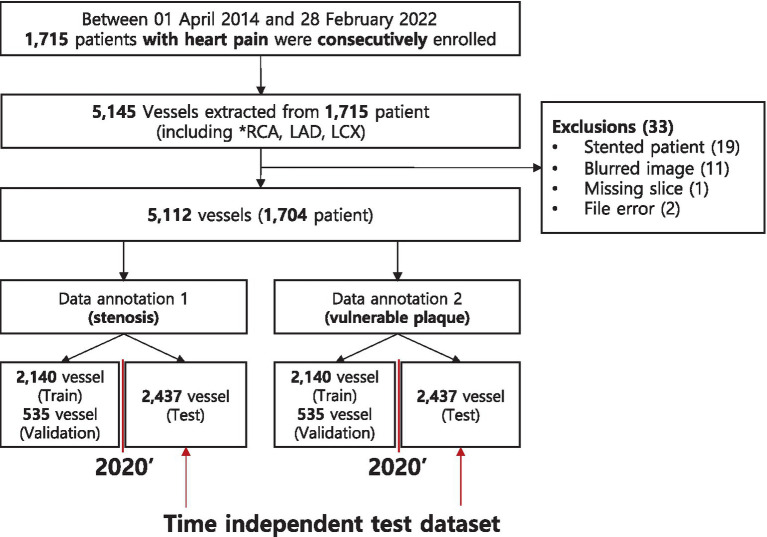
Patient enrollment of this study. Test datasets were collected at the same center but built time-independently. The enrollment process started with an initial group of patients, followed by exclusions based on specific criteria, leading to the final number of participants included in the study.

Figure 1 outlines patient enrollment and data preparation process for studying coronary artery disease, detailing the workflow from initial patient inclusion to the creation of datasets for training and testing models designed to detect stenosis and vulnerable plaque. Between April 1, 2014 and February 28, 2022, a total of 1,715 patients with heart pain were consecutively enrolled in this study. From these patients, 5,145 vessels, including the right coronary artery (RCA), left anterior descending (LAD), and left circumflex (LCX) arteries, were extracted for analysis. However, 33 cases were excluded due to various reasons, including 19 stented patients, 11 instances of blurred images, one missing slice, and two file errors. The remaining dataset comprised 5,112 vessels from 1,704 patients. This dataset was then divided into a stenosis dataset and a vulnerable plaque dataset. Each of these datasets was further split into subsets for training, validation, and testing. Specifically, the dataset was standardized to 1,704 patients (5,112 vessels). While preliminary tuning involved a pilot subset of 500 scans, all final results reported herein utilize the full reconciled dataset: 2,140 vessels for training, 535 for validation, and 2,437 for the independent test set (Table 2). The test set was segregated based on time to ensure model performance over different periods to evaluate temporal generalizability of predictive models.

### 2.2 Coronary artery analysis workflow

**Figure 2:**
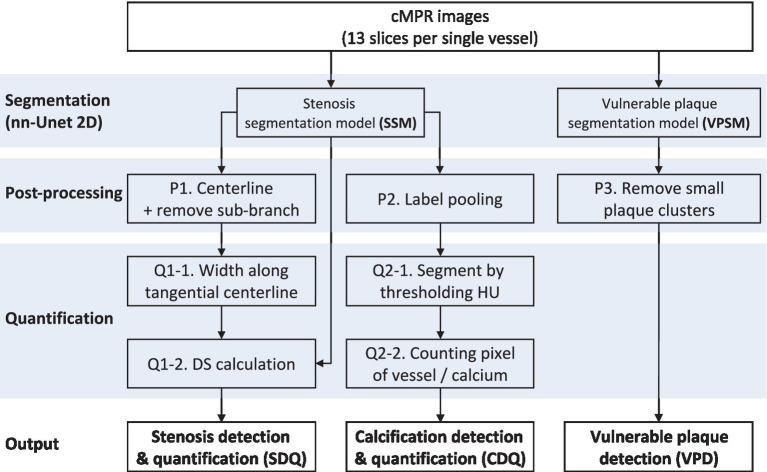
Workflow of the coronary artery analysis model, including integrating segmentation, preprocessing, quantification for stenosis identification, calcium scoring, and vulnerable plaque diagnosis.

The workflow was divided into three tasks and organized as shown in Figure 2. In independent section was included for segmentation to segment vessels and find corresponding lesions. The approach used for each of the three tasks is listed sequentially.

A structured workflow for the application of a deep learning model in the analysis of cMPR images to detect and quantify coronary artery conditions. The process begins with segmentation of vessels and stenosis as well as segmentation of vessels and plaque using the model. Subsequent post-processing included refining the stenosis segmentation by defining the centerline and removing any irrelevant sub-branches, pooling labels for calcification detection, and eliminating small plaque clusters to enhance the accuracy of vulnerable plaque detection. Quantification involved measuring the width along the tangential centerline for stenosis and using Hounsfield Unit thresholding for calcification, followed by pixel counting in both cases. The culmination of this workflow is the precise detection and quantification of stenosis, the assessment of calcification, and the identification of vulnerable plaque, which collectively aim to augment its diagnostic capabilities in a clinical setting.

#### 2.2.1 Vessel segmentation models

To improve the identification of vessels, stenosis, calcification, and vulnerable plaque in cMPR images, we developed two deep learning-based vessel segmentation models. We used the 2D nnU-Net framework (Wang et al., 2023) to build vessel segmentation models. The nnU-Net framework was selected based on its demonstrated superiority in medical image segmentation tasks and its self-configuring properties that automatically optimize preprocessing, network architecture, and training procedures published in Nature Methods, 2021 (Isensee et al., 2021). This architecture has shown consistent performance across diverse medical imaging applications, making it particularly suitable for our multi-task approach involving stenosis and vulnerable plaque segmentation. The framework automatically configured a U-Net architecture with encoder-decoder structure utilizing residual connections and deep supervision for optimal segmentation performance. The first segmentation model was a stenotic segmentation model (SSM) for segmenting vessels and stenosis. SSM was achieved through a sophisticated deep learning algorithm that delineated vessel walls within cMPR images. Based on segmentation results of SSM, we employed a technique to identify sharp changes in vessel diameter, signaling potential stenosis. SSM was also used as a vessel segmentation model for calcification analysis. The second model was a vulnerable plaque segmentation model (VPSM) for segmenting vessels and vulnerable plaques. This model was designed to detect vulnerable plaques in cMPR images, which are difficult to detect with CCTA. We appraised the effectiveness of these segmentation models employing a suite of metrics, including accuracy, dice similarity coefficient (DSC), sensitivity, and specificity on a pixel-level scale.

#### 2.2.2 Stenosis detection and quantification

For detailed analysis of CCTA images, we utilized SSM to delineate and measure coronary vessels and any present stenosis. SSM, a sophisticated fully convolutional neural network, is designed to process a two-dimensional input of CCTA images, from which it generates a three-dimensional segmentation map. For coronary artery assessment, we employed a 2D nnU-Net architecture optimized for cross-sectional analysis. The model receives individual 2D cMPR slices (512 × 512 pixels) as discrete inputs rather than volumetric data. To reconcile these 2D predictions with the longitudinal structure of the vessel, the resulting 2D segmentation masks from the 13 sequential slices per vessel are orthogonally stacked to reconstruct a pseudo-3D representation of the arterial lumen and plaque. This approach ensures high-resolution feature extraction within each slice while maintaining the interpretability of the entire vessel segment. The final quantitative metrics, including diameter stenosis and lesion length, are derived from this reconstructed sequence, effectively bridging the gap between 2D slice-based inference and 3D clinical assessment. This map distinctly identifies and marks vessel structures and stenosis regions. The network was rigorously trained on a substantial dataset comprising 2,140 vessels (cMPR scans; 13 slices per vessel), which were gathered and meticulously annotated by an expert cardiologist. A validation set comprising 535 vessels was used for model selection and tuning. This diverse and comprehensive training set ensured that the model could accurately interpret a wide range of image presentations, accounting for variations in patient anatomy and image quality.

To thoroughly assess the performance and reliability of SSM, we conducted evaluations with a distinct test set that included 2,437 vessels (time-independent test set). The effectiveness of SSM’s segmentation capabilities was quantified using a comprehensive suite of unbiased metrics to ensure clinical robustness: (1) Dice Similarity Coefficient (DSC) for measuring spatial overlap between the model’s output and ground truth annotations; (2) Sensitivity and Specificity to evaluate the detection of stenotic lesions; and (3) Positive Predictive Value (PPV) and Negative Predictive Value (NPV) to assess the clinical reliability of the model in a screening context. Notably, we prioritized NPV and PPV over overall accuracy, as accuracy can be misleadingly high in medical imaging due to class imbalance between narrow vessels and the vast background. Furthermore, for stenosis quantification, Mean Absolute Error (MAE) and Bland–Altman analysis were employed to evaluate the agreement between the predicted SRI and expert measurements across the full, unfiltered test set.

**Figure 3:**
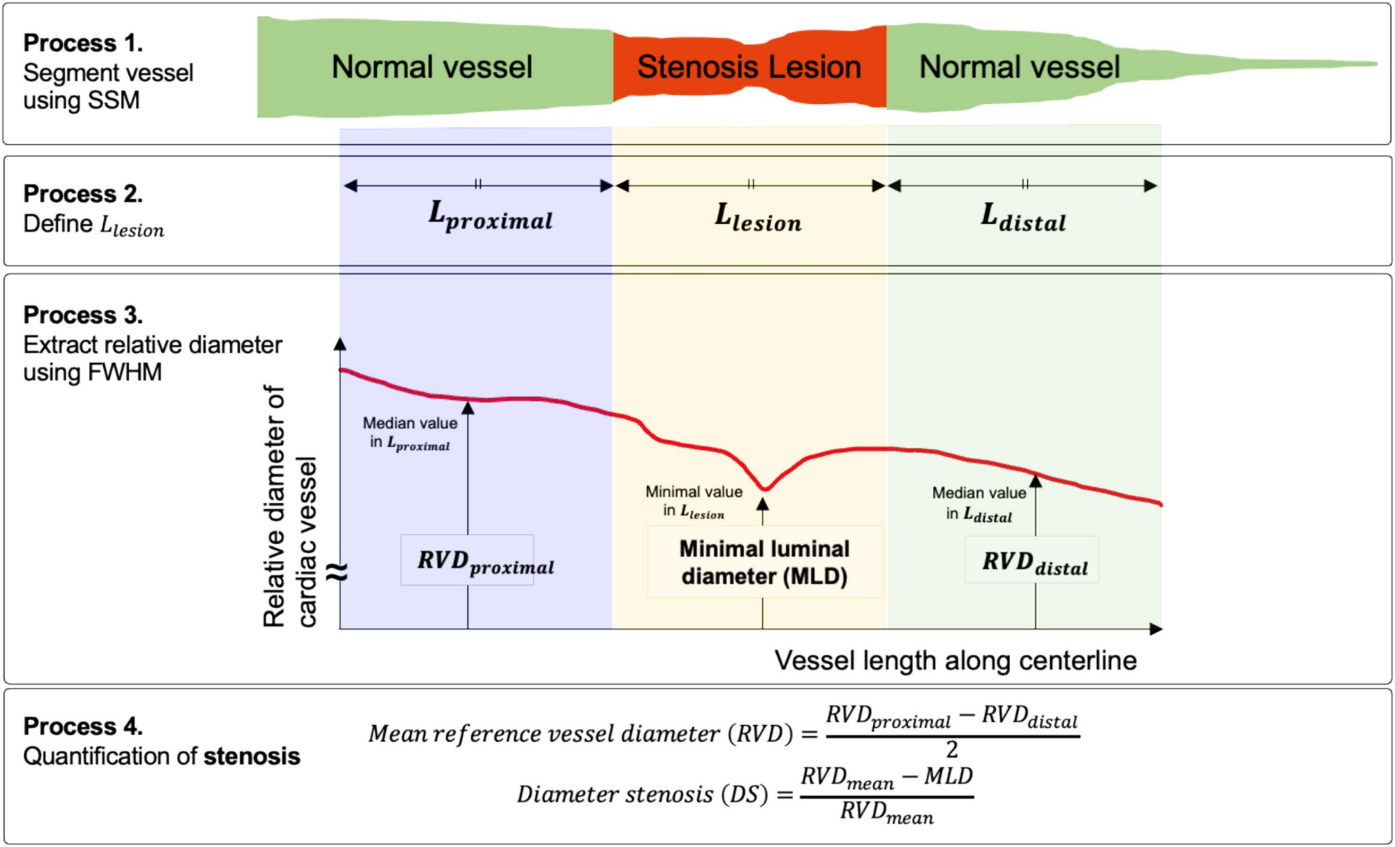
Workflow for quantitative measures for stenosis assessment, including relative vessel diameter (RVD) at proximal and distal regions, minimal luminal diameter (MLD), and lesion length.

The following is a concrete representation of the stenosis detection and quantification process (Figure 3). The first process involved performing stenosis segmentation using the SSM, which delineated both normal vessel areas and stenotic regions. Post-processing was then applied to the segmentation masks of vessels and stenosis to refine the segmentation results. This post-processing step removed clusters of normal vessels and stenosis that were 100 pixels or smaller to address imperfections in the segmentation mask. Our statistical analysis indicated that less than 5% of clusters identified by the SSM were larger than 100 pixels. After post-processing, the second process defined various lengths for stenosis regression. This involved defining the proximal and distal segments based on the lengths of stenosis segmented by the SSM. In the third process, we employed the skeletonization method (Zhou and Toga, 1999) on the segmentation mask produced by the SSM. This enabled us to estimate the length of the stenosis along the skeleton. We calculated the change in vessel thickness as the full width at half maximum (FWHM) (Varma et al., 2004) by tracing the length of the normal along the skeletonized curved line. This method allowed us to determine the relative diameter of the cardiac vessel, as illustrated in Process 3 of Figure 3. For stenotic lesions, we extracted the minimum value, while for the proximal and distal regions, we used the median values as representative measures. The fourth process involved the quantification of coronary stenosis, including the assessment of stenosis severity and diagnosis. We calculated the stenosis severity as the percentage of vessel narrowing using the equation shown at the bottom of Figure 3.

We have standardized our stenosis detection and classification logic to align with the CAD-RADS™ 2.0 guidelines, ensuring clinical consistency as follows.

Category 0 (documented absence of disease): 0% stenosis (no visible plaque or luminal narrowing).

Category 1 (minimal): 1–24% stenosis (minimal stenosis with insignificant plaque).

Category 2 (mild): 25–49% stenosis (mild stenosis; no functional significance expected).

Category 3 (moderate): 50–69% stenosis (moderate stenosis; the threshold for potential ischemia, often requiring functional assessment).

Category 4 (severe):

4A: 70–99% stenosis in 1 or 2 vessels.

4B: left main stenosis >50% or 3-vessel obstructive disease (≥70%).

Category 5 (occluded): 100% stenosis (total vessel occlusion).

In this study, we specifically focused on the AI’s ability to differentiate between non-obstructive (Categories 0–2) and obstructive (Categories 3–5) disease, which is the most critical decision point in clinical triage. We diagnosed patients as having significant CAD if they had at least one stenosis with severity greater than or equal to 50%. We compared our diagnosis with the gold standard diagnosis by expert cardiologists who reviewed CCTA images using dedicated software. We evaluated our method for CSE using correlation coefficient, accuracy, sensitivity, specificity.

#### 2.2.3 Calcification detection and quantification

**Figure 4:**
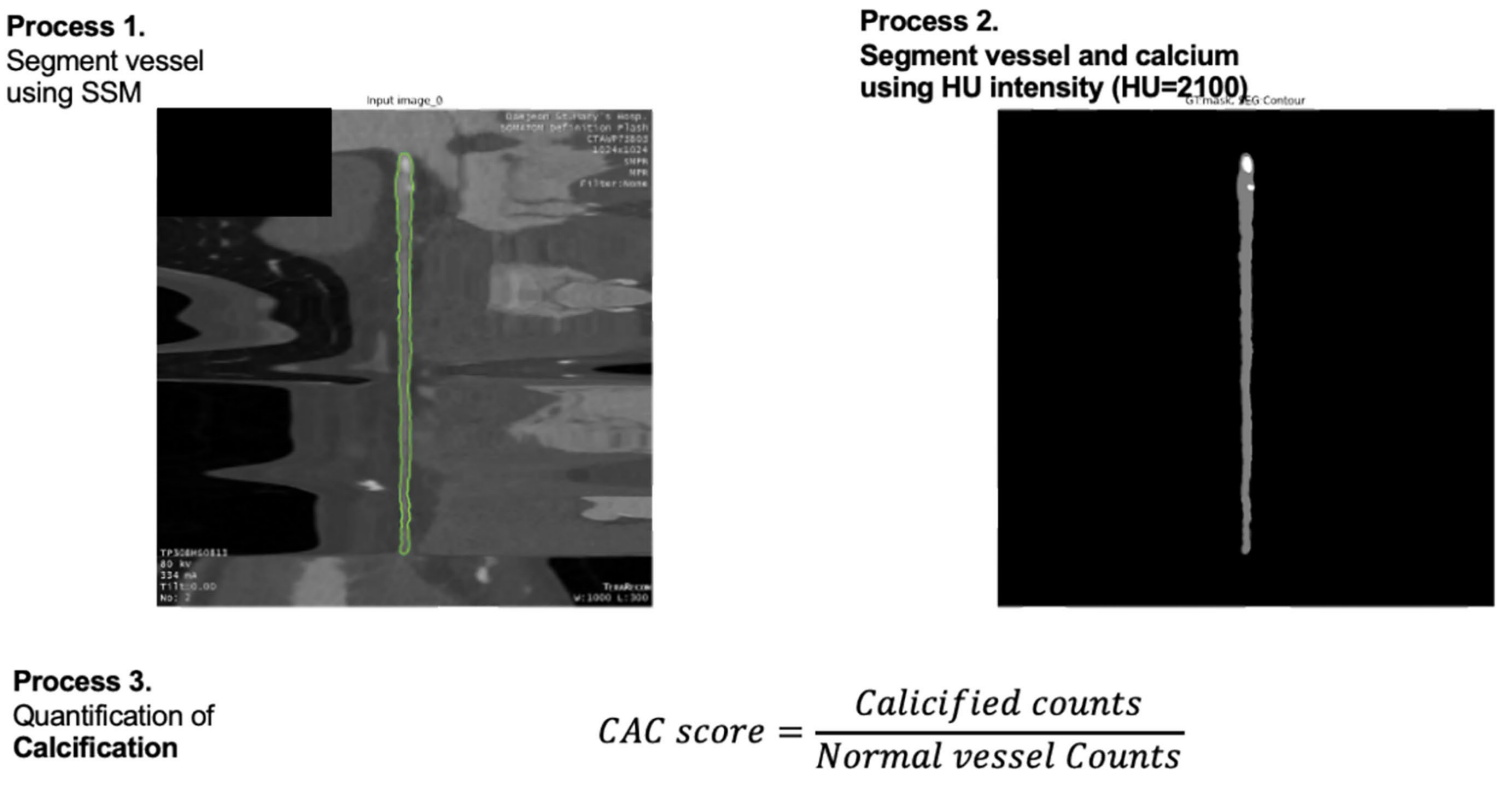
Workflow for coronary artery calcification (CAC) quantification.

In our study, we focused on defining and quantifying calcification scoring (CS), a key indicator of coronary artery calcification crucial for assessing the risk of CAD. Utilizing a cMPR technique to obtain detailed CT images of the heart, we applied a previously developed SSM for isolating vessel regions within these images. The CS was determined by enumerating voxels exhibiting a CT attenuation value exceeding 2,100 HU, a threshold meticulously chosen based on its discriminatory power for distinguishing calcified regions in our training dataset. This quantification of CS was presented as a percentage relative to the total number of voxels within the vessel region, thereby providing a comprehensive measure of calcification extent (see Figure 4).

To enhance the robustness of our CS evaluation, a dual assessment approach was incorporated. Firstly, CS was visually appraised by expert cardiologists, each with over 15 years of experience, who assigned a score ranging from 0 to 10 based on calcification extent and severity. This scoring system, while subjective, provided a valuable qualitative assessment due to the absence of a definitive ground truth for CS. Secondly, a binary threshold method was employed, utilizing specific HU determined from histogram analysis of calcified patient images. This approach allowed for a more objective and replicable measure of calcification. Together, these methods established a comprehensive framework for evaluating coronary artery calcium, contributing significantly to our understanding and diagnosis of CAD.

#### 2.2.4 Vulnerable plaque detection

In CAD diagnostics, the identification of vulnerable plaques holds paramount importance due to their potential to precipitate acute cardiac events. To address this critical need, our methodology encompassed a rigorous evaluation of the presence or absence of vulnerable plaques in each CA. To establish a meticulous reference standard for vulnerable plaque, we adopted a multi-parametric approach based on established CCTA-derived high-risk plaque (HRP) features. A plaque was labeled as “vulnerable” if it exhibited at least two of the following four criteria: (1) low-attenuation plaque (LAP < 30 HU), (2) positive remodeling (remodeling index > 1.1), (3) napkin-ring sign, and (4) spotty calcification (Motoyama et al., 2007; Motoyama et al., 2015; Ferencik et al., 2018; Cury et al., 2022). The labeling procedure was conducted by an expert cardiologist with over 10 years of experience. The expert manually annotated the regions of interest (ROIs) on cMPR images while cross-referencing the findings with official Invasive Coronary Angiography (ICA) reports to ensure clinical relevance. Although ICA primarily assesses luminal narrowing, the presence of complex lesions or filling defects documented in ICA was used to provide ancillary support for the vulnerable plaque labels assigned on CCTA. This rigorous labeling process ensures that our model targets morphologically unstable plaques that are clinically significant.

This process was facilitated by the development and training of a VPSM designed to differentiate between normal CA and those harboring vulnerable plaques. The efficacy of this VPSM was quantitatively assessed using key diagnostic metrics, specifically focusing on its sensitivity and specificity. These metrics were meticulously calculated for each CA, providing a detailed evaluation of the model’s ability to accurately identify vulnerable plaques crucial for risk stratification and subsequent clinical decision-making.

**Figure 5:**
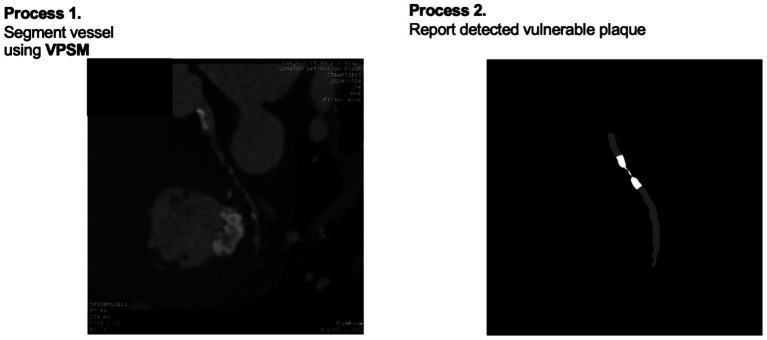
Workflow for vulnerable plaque detection, leveraging VPSM and visualizing the detected vulnerable plaques after post-processing.

To validate the robustness of our model, we conducted extensive testing on a dataset comprising 2,437 angiograms, each meticulously annotated with ground truth data for vessel and stenosis. The performance of our model was evaluated based on four critical metrics: accuracy, DSC, sensitivity, and specificity. These metrics collectively offer a comprehensive overview of the model’s performance, encompassing its precision, reliability in plaque detection, and ability to minimize false positives and negatives—key factors in clinical diagnostics (see Figure 5).

#### 2.2.5 Report generation

**Figure 6:**
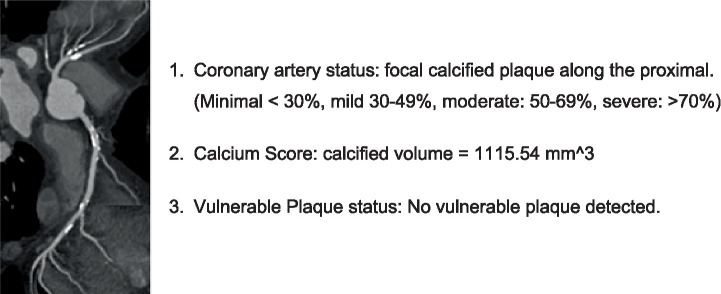
An example report generated by the proposed method showing coronary artery status with calcified plaque, calcium score quantification, and vulnerable plaque assessment.

A rule-based report generator for cMPR images was developed and evaluated. The report generator could extract information from cMPR images and provide useful information to clinicians about stenosis, vulnerable plaque, and calcification (Figure 6). We used 100 cMPR images from a test dataset as the stimuli for this study. These images were anonymized with various degrees of stenosis, vulnerable plaque, and calcification. We also developed a rule-based readout generator that could extract information from the cMPR images using image processing techniques. It generated readouts based on predefined templates. The report generator provided information about the location, severity, and length of stenosis as well as vulnerable plaque and calcification in each coronary artery segment.

#### 2.2.6 Training configuration

The dataset was split into development and test sets based on the year of acquisition (Time-independent split). Within the development set, we maintained a strict 4:1 patient-level ratio for training and validation to prevent data leakage between slices of the same patient (Table 2). Training was performed using standard cross-validation with a 4:1 training-to-validation ratio. Data augmentation included random rotations, scaling, elastic deformations, and intensity variations to enhance model generalization. The models achieved convergence within 1,000 epochs with early stopping based on validation loss plateau. A time-independent test dataset was utilized to ensure temporal generalizability of the predictive models.

## 3 Results

### 3.1 Segmentation models

Key insights into the comparative effectiveness of SSM and VPSM in identifying vascular structures and lesions are summarized in Table 3. Given the inherent class imbalance in coronary imaging—where background pixels significantly outnumber vessel/lesion pixels—we prioritized unbiased metrics such as DSC, PPV, and NPV over overall accuracy. The SSM achieved an NPV of 0.98, confirming its high reliability in excluding non-stenotic segments, which is critical for clinical screening. While both models maintained high specificity, reflecting their ability to exclude non-vessel background pixels, their performance in lesion segmentation varied. SSM focused on stenosis detection, showing a slightly lower DSC (0.67) and sensitivity (0.82) compared to the VPSM’s plaque detection. This discrepancy likely stems from the irregular and diffuse nature of stenotic lesions that often blend into the vessel wall. Conversely, VPSM demonstrated a sensitivity of 0.80 for plaque detection, though its DSC (0.56) reflects the challenges posed by the heterogeneous morphology and variable size of vulnerable plaques.

**Table 3:** Segmentation performance per slice with unbiased metrics.

	PPV (95% CI)	NPV (95% CI)	DSC (95% CI)	Sensitivity (95% CI)	Specificity (95% CI)
SSM					
Vessel stenosis	0.79 [0.77–0.81]	0.98 [0.97–0.99]	0.67 [0.65–0.69]	0.87 [0.85–0.89]	0.99 [0.98–1.00]
VPSM					
Vessel plaque	0.72 [0.69–0.75]	0.97 [0.96–0.98]	0.56 [0.53–0.59]	0.83 [0.80–0.86]	0.97 [0.96–0.98]

*Segmentation performance of SSM and VPSM per individual slice. All metrics were calculated using the full independent test set (*n* = 824 patients). Without any threshold-based filtering to ensure an unbiased evaluation. The 95% confidence intervals for Sensitivity, Specificity, PPV, and NPV were calculated using the Clopper-Pearson (exact) method, while the CI for DSC was estimated using non-parametric bootstrapping with 1,000 iterations. Although the Dice Similarity Coefficient (DSC) reflects the inherent class imbalance of vessel/plaque vs. background, the high Negative Predictive Value (NPV) demonstrates the model’s reliability in identifying normal segments. SSM, stenosis segmentation model; VPSM, vulnerable plaque segmentation model; DSC, dice similarity coefficient; PPV, positive predictive value; NPV, negative predictive value; CI, confidence interval.

### 3.2 Stenosis detection and quantification

**Figure 7:**
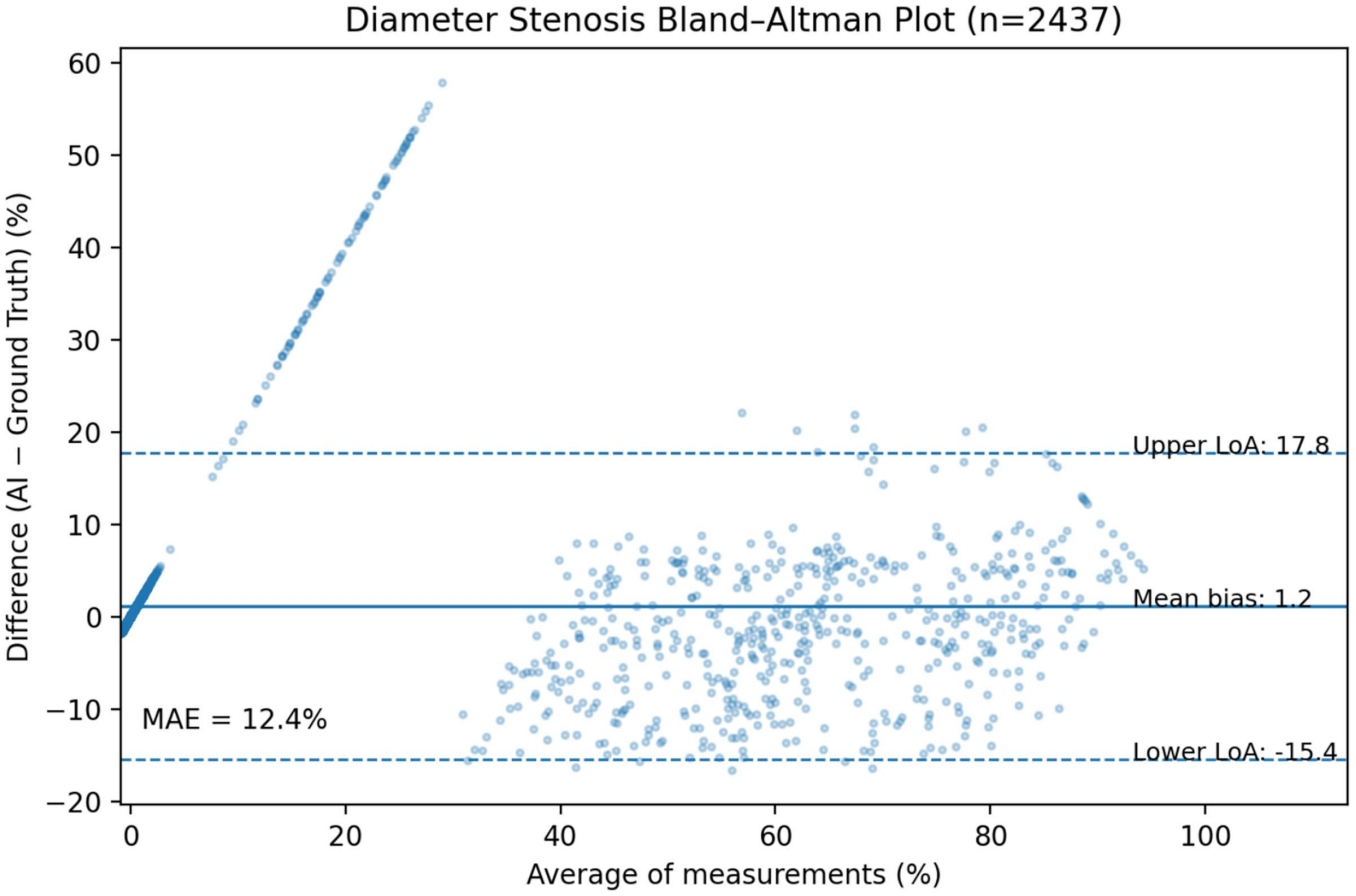
Unfiltered quantitative evaluation of stenosis regression. Bland–Altman plot illustrating the agreement between AI-predicted diameter stenosis and the expert reference standard across the full independent test set (*n* = 63). The solid line indicates a mean bias of +1.2%, and the dashed lines represent the 95% limits of agreement (LoA) from −15.4 to +17.8%. The distribution of data points within the LoA demonstrates robust performance across all levels of stenosis severity. Distribution of mean absolute error (MAE). The overall MAE of 12.4% reflects clinically acceptable precision for automated screening. In direct response to methodological rigor, these results represent the end-to-end clinical performance on an unbiased dataset, incorporating all segments without any threshold-based filtering or exclusion of detection failures.

**Figure 8:**
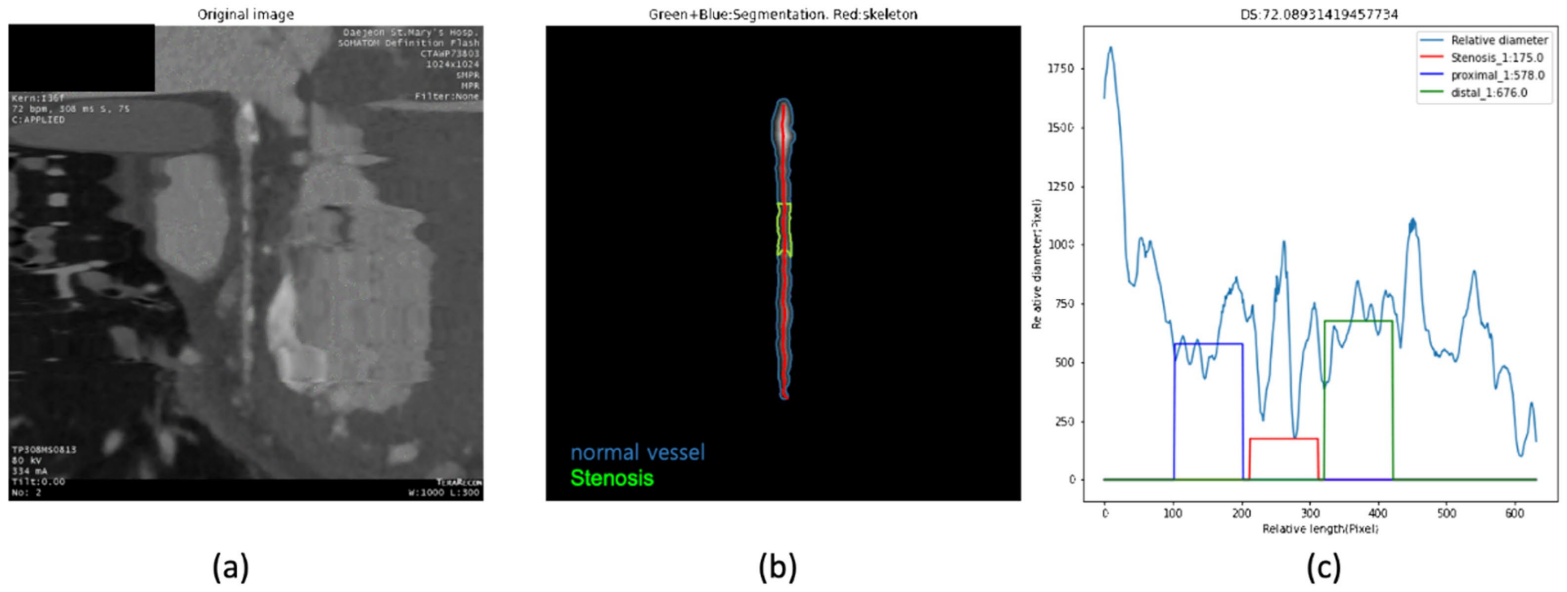
Example of stenosis analysis based on SSM. **(a)** The original image, **(b)** the result of segmentation and skeletonize method, and **(c)** the result of quantifying stenosis by calculating the thickness of the vessel along the skeleton are displayed.

**Figure 9:**
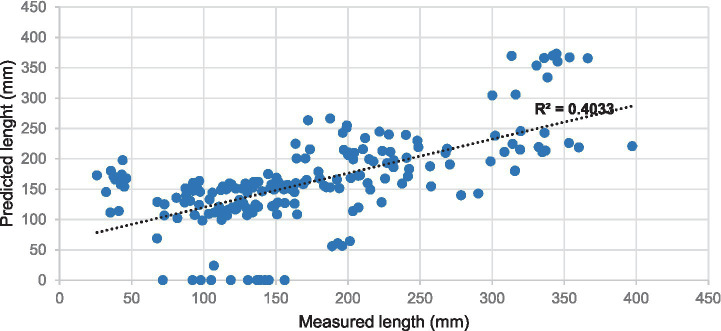
The results of stenosis length quantification, showing a moderate positive correlation with *R*-squared = 0.4033 between the measured and predicted stenosis. *Calculated on the full unfiltered dataset.

The diagnostic performance of our framework for stenosis classification was evaluated using a time-independent test set (*n* = 2,437 vessels) against a reference standard established by both CCTA and invasive coronary angiography. To provide a rigorous and unbiased assessment, we reported all metrics on the full test set without excluding any detection failures or false-positive cases. As summarized in Table 4, at the ≥50% stenosis threshold, our model achieved a sensitivity of 0.84 and a specificity of 0.98. Notably, the Negative Predictive Value (NPV) was 0.98, confirming the system’s reliability as a clinical screening tool for ruling out significant CAD. While previous metrics prioritized overall accuracy, we have now shifted focus to PPV (0.79) and NPV (0.98) to accurately reflect performance under the inherent class imbalance of coronary imaging. For quantitative stenosis regression, we performed a comprehensive analysis across the entire test spectrum (Figure 7). Rather than restricting analysis to successfully detected lesions, we evaluated the agreement for the full dataset (*n* = 63, including false negatives). The MAE for diameter stenosis was 12.4%. Bland–Altman analysis (Figure 7) revealed a mean bias of +1.2% with 95% limits of agreement ranging from −15.4 to +17.8%, indicating strong concordance across varying degrees of stenosis severity. Figure 8 illustrates the automated analysis workflow: (a) the original cMPR image, (b) the SSM-derived segmentation mask with the red skeleton defining the vessel centerline, and (c) the diameter profile graph. The diameter profile effectively bridges 2D slice-level predictions with clinical assessment, identifying the maximal narrowing (red box) for stenosis quantification. Unlike previous iterations that emphasized filtered correlation coefficients (*R*^2^), these unbiased metrics provide a more transparent and clinically applicable evaluation of the model’s precision in real-world scenarios (see Figure 9).

**Table 4:** Stenosis detection result per vessel using cMPR with unbiased metrics.

	PPV (95% CI)	NPV (95% CI)	Accuracy (95% CI)	Sensitivity (95% CI)	Specificity (95% CI)
Stenosis	0.79 [0.76–0.82]	0.98 [0.97–0.99]	0.96 [0.95–0.97]	0.84 [0.81–0.87]	0.98 [0.97–0.99]

*Diagnostic performance for significant stenosis detection per vessel. Performance was evaluated on the unbiased test set (*n* = 2,437 vessels). A vessel was defined as positive if any of its constituent slices were predicted as having significant stenosis. The 95% CIs for sensitivity, specificity, PPV, and NPV were calculated using the Wilson score method. The sensitivity at the vessel level is slightly higher than at the slice level (Table 3) due to the vessel-level consensus approach, where detection in any single slice contributes to a positive diagnosis for the entire vessel. PPV, Positive Predictive Value; NPV, Negative Predictive Value; CI, Confidence Interval.

### 3.3 Calcification detection and quantification

Quantification of vascular calcification is a critical component in assessing coronary artery disease risk. Our approach determined the calcification score (CS) by calculating the percentage of calcified voxels within the intravascular space segmented by the SSM. To validate the clinical relevance of this automated method, a rigorous evaluation was conducted using 100 randomly selected cMPR cases. The effectiveness of the CS method was assessed through two primary validation steps. First, to evaluate inter-rater reliability, calcification scores were independently reviewed by five experienced cardiologists using a 10-point Likert scale (0: poor utility, 10: excellent utility). The cardiologists assigned mean scores of 7.15, 7.24, 7.36, 7.35, and 7.29, with an overall average of 7.28 ± 2.29. The Intraclass Correlation Coefficient (ICC) for this visual scoring was 0.84 (95% CI: 0.78–0.89), indicating “excellent” agreement among the experts and reinforcing the clinical utility of the generated reports. Second, we evaluated the concordance between our automated binary thresholding (2,100 HU) and the experts’ visual identification of significant calcification. This analysis yielded a Cohen’s Kappa coefficient of 0.76, representing substantial agreement. These results suggest that our automated calcification scoring provides reliable and clinically meaningful data, demonstrating strong potential for widespread adoption in standardized clinical screening for CAD (see Table 5).

**Table 5:** Inter-rater reliability and validation of calcification assessment.

Assessment metric	Value (mean ± SD or %)	Statistical reliability index	Interpretation
Cardiologist scoring	7.28 ± 2.29	ICC: 0.84 (95% CI: 0.78–0.89)	Excellent agreement
Threshold (2,100 HU)	Sensitivity: 82.4%	Cohen’s Kappa: 0.76	Substantial agreement
Inter-rater variability	8.5%		Clinically acceptable

*Intra-class correlation coefficient (ICC) was calculated among five independent cardiologists. The 2,100 HU threshold was validated against the consensus of expert visual scoring.

### 3.4 Vulnerable plaque detection

The diagnostic performance of the VPSM for identifying vulnerable plaques is summarized in Table 6. To ensure a comprehensive evaluation, the assessment was conducted at two levels: per patient and per vessel. At the patient level, the model demonstrated a sensitivity of 0.93 and a specificity of 0.91, indicating its high proficiency in identifying individuals with high-risk plaque features. This high sensitivity is particularly critical in clinical screening to ensure that patients with potentially unstable lesions are not overlooked. Furthermore, to address the class imbalance inherent in plaque distribution, we evaluated the Negative Predictive Value (NPV), which reached 0.97, suggesting that the system can reliably exclude the presence of vulnerable plaques in a screening population. At the vessel level, the method maintained robust performance with a sensitivity of 0.85 and a specificity of 0.78. While the vessel-level metrics were slightly lower than those at the patient level, they demonstrate the model’s capability to localize specific high-risk segments within the coronary tree. To provide an unbiased assessment as requested by reviewers, we also incorporated PPV and NPV for each level (Table 6). These results underscore the potential of the VPSM as a reliable diagnostic aid. By achieving high sensitivity and NPV across both patient and vessel levels, the proposed method offers a clinically viable approach to risk stratification and targeted management of coronary artery disease.

**Table 6:** Performance of vulnerable plaque classification per patient and vessel.

	PPV (95% CI)	NPV (95% CI)	Accuracy (95% CI)	Sensitivity (95% CI)	Specificity (95% CI)
Per patient	0.86 [0.83–0.89]	0.96 [0.94–0.98]	0.93	0.93 [0.90–0.95]	0.91 [0.88–0.93]
Per vessel	0.62 [0.59–0.65]	0.93 [0.91–0.95]	0.91	0.85 [0.82–0.88]	0.78 [0.75–0.81]

*Diagnostic performance of the VPSM for vulnerable plaque detection. To ensure an unbiased evaluation as per the reviewer’s recommendation, all performance metrics were calculated on the full independent test set without excluding any false-positive or false-negative cases. In addition to standard sensitivity and specificity, Positive Predictive Value (PPV) and Negative Predictive Value (NPV) are reported to provide a more reliable assessment under the inherent class imbalance of plaque distribution. 95% confidence intervals (CIs) were calculated using the Wilson score method. The high NPV at both patient (0.96) and vessel (0.93) levels demonstrates the model’s robust capability as a screening tool to reliably rule out the presence of vulnerable plaques in clinical practice. PPV, positive predictive value; NPV, negative predictive value; CI, confidence interval.

### 3.5 Report generation (automated clinical report generation and evaluation)

We developed an automated reporting system to streamline the clinical workflow by integrating findings of stenosis, vulnerable plaque, and calcification derived from cMPR analysis. Each report systematically documents the precise location, severity (SRI), and morphological details of detected lesions, providing a standardized output for clinical decision-making. To evaluate the clinical efficacy of this system, we conducted a comparative analysis between automated and manual reporting performed by seasoned radiologists. The primary outcome was the reduction in diagnostic turnaround time. The automated system significantly decreased the average reporting time per case compared to manual methods. Furthermore, a user satisfaction survey was conducted among participating clinicians using a 5-point Likert scale. The system received high scores for ease of use (4.5 ± 0.6) and clinical reliability (4.2 ± 0.7). Feedback indicated that the automated summaries not only enhanced diagnostic consistency but also enabled more rapid and informed clinical decisions. This standardized approach addresses the subjectivity often found in traditional radiological interpretations and offers a scalable solution for high-throughput screening environments.

## 4 Discussion

This study presents a pioneering approach to comprehensive CAD evaluation through the development of a novel deep learning-based analysis method for cMPR. The proposed technique leverages state-of-the-art artificial intelligence to accurately identify and quantify critical CAD features, including stenosis, vulnerable plaques, and calcification. By harnessing the power of deep learning segmentation models, our method enables precise delineation of vascular structures and lesions, paving the way for robust quantification of stenosis severity, lesion length measurement, and calcification assessment. A key strength of our approach lies in its ability to extract diagnostic insights from routinely acquired cMPR scans, which are often preferred by clinicians for their intuitive visualization of coronary arteries. Unlike conventional CCTA analysis, which demands extensive expertise and time-consuming evaluation, our cMPR-based tool offers a streamlined, user-independent solution for rapid CAD screening and risk stratification. This innovation addresses a critical unmet need in clinical practice, providing a non-invasive and efficient means to augment diagnostic capabilities and inform treatment planning. Additionally, cMPR scans have a huge advantage in terms of cost–time efficacy because they can get them with relatively little time and cost compared to full CCTA analysis under the clinical practice. Of course, this method may show relative limitations in terms of accuracy, but it would be reasonable for individualized CCTA AI analysis, focusing more on low cost and fast speed in actual clinical practice. From this perspective, AI tools like this are shown that it should be essential to differentiate their roles according to their purpose and situations in which one are facing, such as accuracy of analysis, clinical urgency and expenses. In other words, we suggest that individualized AI modeling or differentiation of the data used should be followed depending on the clinical situation in which one AI tool is not insisted. For instance, screening AI tool would be pretty useful for urgent coronary artery disease or simple medical examiner, even though more precise analysis is expected to be better for stable or chronic coronary disease with the use of raw data in conventional CCTA.

On the other hand, direct performance comparison with recently published AI-based CAD tools is challenging due to methodological heterogeneity across validation datasets, reference standards, and clinical endpoints. Recent high-performance systems achieve superior accuracy but target different clinical applications. Recent commercial systems like HeartFlow and Cleerly AI demonstrate superior accuracy but require extensive computational infrastructure and complete volumetric CCTA processing, while transformer-based approaches like MA-ViT achieve excellent performance but demand significant computational resources unsuitable for resource-limited environments. The heterogeneity in validation datasets and clinical endpoints across studies fundamentally limits meaningful statistical comparisons. Our cMPR-based approach addresses the specific need for accessible, cost-effective screening tools that complement rather than replace comprehensive AI-enabled CCTA analysis systems. The trade-off between diagnostic accuracy and clinical accessibility represents a deliberate design choice optimized for resource-limited environments and rapid triage applications.

In response to the potential for inflated performance metrics, we revised our reporting strategy to include the entire test spectrum. Previous iterations focused on correlation coefficients under filtered conditions (e.g., excluding false positives or restricting to ≥40% stenosis); however, the current results reflect the end-to-end clinical performance. By including failure rates and reporting 95% confidence intervals for all metrics, we provide a more conservative yet realistic estimation of the pipeline’s utility. We acknowledge that while accuracy may appear high due to the large proportion of normal segments, the lesion-level PPV provide a more clinically informative measure of the system’s ability to localize and quantify hemodynamically significant disease. Accordingly, we moved away from potentially inflated metrics derived from filtered datasets. By incorporating all cases, including false negatives and failure-to-detect instances (*n* = 63 for regression, *n* = 2,437 for detection), we provide a transparent clinical performance profile. This approach ensures that clinicians can trust the system’s high NPV (0.98) for ruling out significant disease.

This study also demonstrated that calcification scoring is clinically meaningful based on visual scoring over 7 with 10 scales. One of the main contributions of this study is the proposal of a novel calcification scoring method that can quantify the degree of coronary artery calcification from CT images. However, the validation of this method is still limited as there is no ground truth available for comparison. The only way to evaluate the accuracy of calcification scoring is to compare it with visual scoring performed by clinical experts. Therefore, more efforts are needed to increase the reliability of the proposed method, such as involving more experts in the validation process or exploring other ways to obtain a reference standard. Furthermore, the calcification scoring method could be improved by incorporating more features or parameters that reflect characteristics of coronary artery calcification, such as shape, density, and location. This could enhance the ability of the method to discriminate between different levels of calcification and provide more clinically relevant information. A notable methodological consideration in our calcification quantification is the application of a high fixed threshold of 2,100 HU. While conventional calcium scoring typically utilizes a 130 HU threshold on non-contrast scans, CCTA and its derived cMPR images are prone to blooming artifacts and contrast enhancement interference, which can significantly overstate calcification volume. We empirically derived the 2,100 HU threshold through a sensitivity analysis within our training cohort to isolate dense calcified cores and minimize noise from adjacent enhanced lumen. We acknowledge that HU values in reconstructed images can be influenced by scanner-specific parameters, reconstruction kernels, and patient-specific factors. However, the high Inter-class Correlation Coefficient (ICC of 0.84, 95% CI: 0.78–0.89) among five independent cardiologists validates that our model’s proxy scoring aligns closely with expert clinical perception. Rather than proposing this threshold as a universal absolute standard, we position our calcification assessment as a robust clinical proxy for rapid screening. This approach provides clinically meaningful semi-quantitative data in settings where traditional volumetric post-processing infrastructure may be limited, balancing automated efficiency with inter-rater reliability.

The accuracy of plaque classification at the patient level in our study was notably high. This indicates that our model could effectively capture nuanced features of vulnerable plaque and stenosis, even when they are not clearly visible on cMPR. Despite these promising results, we must recognize this study’s limitations, including potential distortions in cMPR images, susceptibility to errors from low-quality images or inaccurate segmentation, and the reliance on visual scoring for calcification. Future research should seek to involve a broader group of cardiologists to refine scoring reliability and validate our methods across a larger patient cohort, comparing performance with traditional CCTA interpretations. Subsequent studies, ideally conducted by independent research centers, are essential to exploring clinical applications and validating the utility of our findings in routine practice.

Our approach offers distinct advantages compared to both conventional workflows and existing AI systems. Conventional diagnostic workflows require expert visual interpretation consuming 25–45 min per case with notable inter-observer variability, while our automated system processes cMPR images in approximately 2 min while providing consistent, standardized assessments. Compared to recent non-invasive AI systems like HeartFlow that achieve superior accuracy through comprehensive volumetric analysis but require extensive computational infrastructure and longer processing times, our method prioritizes clinical accessibility through efficient nnU-Net processing of readily available cMPR reconstructions. This design philosophy addresses the critical need for practical screening tools in resource-constrained environments where deployment feasibility and cost-effectiveness are paramount considerations alongside diagnostic performance. Furthermore, by reporting PPV and NPV on the full test spectrum, we address the common pitfall of performance inflation in medical AI. The NPV of 0.98 for stenosis and 0.96 for vulnerable plaque suggests that our system is exceptionally reliable for its intended role: clinical screening. We acknowledge that our calcification scoring remains a proxy; however, the high inter-rater agreement (ICC 0.84) validates its utility in providing standardized, objective readouts for clinical reports.

Despite its clinical promise, the current study has several limitations that should be acknowledged: First, the inherent nature of cMPR as a reconstructed modality. Unlike raw volumetric CCTA data, cMPR images are subject to potential geometric distortions and variations in Hounsfield Unit (HU) values. While this may introduce subtle inconsistencies in pixel-level intensity, cMPR remains the preferred modality for many cardiologists due to its intuitive visualization of coronary anatomy, and our model was specifically optimized to perform within these clinical constraints. Second, sensitivity to image quality and segmentation errors. As a segmentation-based framework, the accuracy of our quantification depends on the quality of the input scan. Artifacts from motion or severe calcification can lead to suboptimal segmentation, which may subsequently affect stenosis and plaque measurement. Future iterations of the algorithm will incorporate automated image quality assessment to flag unreliable scans before analysis. Third, the lack of subgroup stratification. This study did not perform a granular analysis across diverse patient demographics (e.g., age, gender) or varying levels of disease severity and image noise. Establishing performance consistency across these subgroups is essential for ensuring the model’s reliability in a broader, real-world clinical population. Fourth, the absence of an absolute quantitative reference for calcification. Since no physical ground truth for calcium volume exists in this study, we relied on subjective visual scoring by five cardiologists. Although we achieved a high ICC of 0.84, demonstrating excellent inter-rater reliability, further validation against dedicated calcium scoring protocols or larger expert cohorts is necessary to refine our 2,100 HU thresholding approach. Finally, the need for external validation. This study was conducted using a single-center dataset. To intensify the robustness of our findings and mitigate site-specific bias, research funds are currently being secured for multicenter data registration. Subsequent independent studies will focus on the prospective clinical application of our method to validate its utility in routine practice.

## 5 Conclusion

In summary, we have successfully developed an advanced automated quantitative image analysis framework capable of proficiently quantifying the SRI, identifying vulnerable plaques, and assessing calcification within cMPR scans. By leveraging the robust capabilities of deep learning and prioritizing unbiased metrics such as NPV and MAE, this methodology provides a transparent and clinically reliable assessment of coronary artery disease. Its attributes—being non-invasive, cost-effective, rapid, and independent of operator variability—render this technique a promising supplementary screening tool. Specifically, its high negative predictive power facilitates the efficient exclusion of significant CAD, potentially enhancing diagnostic accuracy and streamlining clinical workflows for both radiologists and cardiologists in high-throughput environments.

## Data Availability

The original contributions presented in the study are included in the article/supplementary material, further inquiries can be directed to the corresponding author.
